# ASAS-NANP symposium: mathematical modeling in animal nutrition: Model Transfer and Application in Livestock Production Systems

**DOI:** 10.1093/jas/skag308

**Published:** 2026-09-30

**Authors:** Hector M Menendez, Jordan M Adams, Karun Kaniyamattam, Luis O Tedeschi

**Affiliations:** Department of Animal Science, South Dakota State University, Rapid City, SD 57703, United States; Department of Animal Science, Texas A&M University, College Station, TX 77843-2471, United States; Department of Animal Science, Texas A&M University, College Station, TX 77843-2471, United States; Department of Animal Science, Texas A&M University, College Station, TX 77843-2471, United States

**Keywords:** translational models, model predictive control, equitable profit, food system transformation, feed management

## Abstract

Mathematical models have long supported nutrition management in livestock systems, progressing from early National Research Council empirical tables to complex mechanistic frameworks such as the Ruminant Nutrition System. What is less documented is how models are *operationalized* in commercial practice, particularly as data streams from precision livestock technologies, remote sensing, and other Internet of Things technologies are increasingly hybridized into existing modeling frameworks. A prevailing assumption is that greater sensing, automation, or algorithmic complexity will naturally translate into better decisions; however, adoption and impact in commercial systems remain uneven. To examine how models are actually used under real-world constraints, we conducted a literature review and semi-structured interviews (*n* = 11) with livestock producers, technology developers, consultants, and value-chain actors working at the interface of biology, data, models, and farm decision-making. Six cross-cutting themes emerged: 1) model augmentation of systems, 2) model predictive control (MPC), 3) human centered adoption, 4) technology fit, 5) data bottlenecks, and 6) data enlightened value chain. Collectively, these insights re-center nutrition as the integrating discipline for productivity, welfare, and sustainability, and emphasize MPC and open, interoperable data pipelines, as prerequisites for moving beyond pilots toward durable adoption. Interviewees consistently emphasized that meaningful impact depends on aligning biological realism, software engineering, and value-chain incentives so that models support actionable decisions under commercial constraints, rather than pursuing automation for its own sake.

## Teaser

Model transfer has the greatest impact in livestock systems when biology, data, and decision models are integrated into human workflows. Adoption accelerates when these models fit daily farm operations and return clear economic value across the food system.

## Introduction

### Livestock nutrition model history and current state

Long before digital agriculture and precision livestock technologies, mathematical models were already shaping decisions in livestock production systems. Beginning in the 1940s, the National Research Council (NRC) developed empirical models for estimating livestock energy and nutrient requirements (NRC 1944a, 1944b, 1945a, 1945b, 1945c, 1949, 2011) laying the groundwork for modern diet formulation and nutrition models. Over subsequent decades, these empirically derived models evolved alongside advances in animal physiology, feed characterization, and computational capacity, enabling the development of more mechanistic and integrative models that support modern livestock production systems (Stephens 2021; Tedeschi et al. 2023; Tedeschi and Menendez 2025, 2026). Building on these foundational and nutrition models, industry-driven models emerged to evaluate production efficiency, environmental sustainability, and economic viability, with sensor- and data-enabled technologies accelerating the adoption and practical application of model-based decision support across entire livestock production systems (Condotta and Tedeschi 2026). Livestock systems represent some of the most complex and information-limited production environments in animal agriculture (Tedeschi 2019), which can generally be categorized into confined and extensive systems (Menendez et al. 2022). These systems vary in their use of mathematical nutrition models for livestock, for which the National Animal Nutrition Program (NANP) has been the global leader of bringing comprehensive reviews, training, and resources (Table 1).

**Table 1:** Model resources for different livestock species.

Species	Resource/model type	Citation
**Swine**	Mechanistic-Real-Time Review Resource Review Mechanistic/Agent Based Model	Brossard et al. (2023); Gaillard et al. (2020); NRC (2012); Pettigrew and Esnaola (2001); Rahimifar et al. (2026)
**Poultry**	Review Resource Review	Gous (2007); NASEM (2026); Oviedo-Rondón (2025)
**Small ruminant**	Mechanistic (SRNS) Resource Review Review	Cannas et al. (2007, 2010); NRC (2007a); Pulina et al. (2013); Tedeschi et al. (2019)
**Dairy**	Review Review Dynamic/Mechanistic Life Cycle Assessment Resource Review	Hanigan et al. (2006); Kebreab et al. (2009); Li et al. (2018); Naranjo et al. (2020); NASEM (2021); White et al. (2017)
**Horse**	Mechanistic Review Empirical	Cordero (2013); Tylutki (2011);NRC (2007b)
**Beef**	Resource Resource	NASEM (2016); Tedeschi and Fox (2020)
**Aquaculture**	Dynamic Energy Budget Resource Bioenergetic	Karthikeyan et al. (2026); NRC (2011); Stavrakidis-Zachou et al. (2025)

Mathematical nutrition and production models developed across beef, dairy, swine, poultry, sheep, goat, and aquaculture systems increasingly depend on continuous animal- and environment-level data streams to improve prediction accuracy, biological interpretation, and decision support. Maximizing the utility of such data streams aligns closely with the goals of the NC1211 Precision Management of Animals for Improved Care, Health, and Well Being of Livestock and Poultry Multi-State Project, which integrates sensing technologies, data science, artificial intelligence (AI), and digital infrastructure to support real-time livestock management across species and production systems. More recently, the transfer of mathematical nutrition models has been furthered through the NANP Feed Management committee in partnership with the Natural Resources Conservation Service to assist producers in utilizing these tools and resources. Thus, animal management has evolved from analogue data collection methods (notepad and pencil) toward integrated precision livestock farming systems that combine vision-based technologies, wearable and identification sensors, environmental and air-quality monitoring systems, AI, and interoperable digital platforms to support nutrition, health, welfare, behavior, and management decisions across livestock industries (Berckmans 2017; Menendez et al. 2022; Condotta and Tedeschi 2026).

In the last two decades, Agriculture 4.0 has accelerated beyond intensive animal feeding operations to extensive systems with the deployment of sensors, including collars, ear tags, and scales, alongside virtual fencing and remote sensing technologies such as satellites and drones (unmanned aerial vehicles or autonomous ground vehicles), offering multi-scale, high-frequency observations of animals, forages, and their environments. A critical point is that the efficiencies gained at such extensive phases carry over to downstream confined feeding sectors, packers, and the consumer (Menendez and Tedeschi 2020). Ultimately, the goals are the same across confined and extensive systems, maximizing livestock gains, while decreasing costs through precision nutrient management. The benefits for producers and stakeholders from mathematical models enabled by technology are promising, yet adoption remains uneven, where labor is scarce, margins are tight, and management decisions must be made across large confined production systems and animal classes or heterogeneous grazing landscapes.

### Models and technology are not the gap

Importantly, this adoption gap is not driven by a lack of models or tools for generating data streams to inform models, but by the difficulty of integrating these tools into real decision systems that operate under commercial constraints. Large-scale model transfer in livestock systems, at a speed and scale not previously possible, is needed to put farmers first by translating biological, economic, and environmental data into practical decisions. These decisions include improving profitability, enabling market access through credible measurement, strengthening biosecurity through early detection, regenerating soils via adaptive grazing, and improving food quality by linking on-farm nutrition to downstream health outcomes (USDA 2025).

To contextualize the current state of mathematical nutrition model adoption and to examine how models are transferred from developers to users in practice, we synthesized insights from interviews with industry leaders and academics who apply mathematical, nutrition, livestock, and environmental models in real-world livestock systems. The insights gained from years of experience are likely to help others seeking to deploy mathematical nutrition models to avoid critical mistakes, identify opportunities and challenges, and achieve long-term success. Thus, the objective was to review the current applications, challenges, and potential of livestock nutrition models within the context of real-world application through interviews with experienced industry professionals.

## Materials and methods

### Stakeholder interviews

We conducted a standard literature review of mathematical nutrition models used for livestock production in confined and extensive systems and linked it to the growing body of the NANP modeling committee publications (Tedeschi and Menendez 2026). The synthesis of the overarching state of mathematical livestock nutrition models and NANP collection was then used to create a framework in the form of five key questions for industry professionals that use mathematical models daily. Semi-structured interviews (*n* = 11) were conducted with industry leaders actively engaged in the application of mathematical, nutrition, livestock, and environmental models within real-world decision-making contexts. The objective of the interviews was to extract insight into how models are used, interpreted, and integrated into operational decision systems under biological, economic, and organizational constraints, rather than to evaluate or promote specific technologies or commercial products.

Interviewees represented a cross-section of livestock producers, technology developers, consultants, and value-chain actors, including Nick Jorgensen (Jorgensen Land & Cattle™), Jeff Clark (Sustainable Livestock Analytics™), Shane White and David Smith (CERES Tag™), Andrew Holland and Virgilio Ambriz-Vilchis (Ontrak Nutrition and Ontrak Labs™), Reid Hensen (Celium Group™), Jim Eckberg (Edge Dairy Farmer Cooperative™), Meghan Filbert (Nofence™), Theo Beaumont (Halter™), Heiner Lehr (Beak Analytics™), Daniel Berckmans (BioRICS™), and Ashley Sweeting (AgriScent™). An initial email was sent out to advertise the interview opportunity for the current paper and distributed through animal science networks to industry stakeholders. Participants were purposefully selected based on their leadership roles and demonstrated experience applying modeling frameworks, AI, and precision livestock technologies in commercial settings. Each participant completed a single interview lasting approximately 30–60 min. Interviews were conducted virtually and followed a standardized protocol consisting of five broad, open-ended questions to ensure consistency across interviews while allowing flexibility for participants to elaborate based on their specific expertise and operational context. Questions were asked in rotation among co-authors (*n* = 4) to interviewees.

### Interview questions

The following prompts guided all interviews:

1. Organizational role and mission
  Please describe your organization’s primary goals and mission, and briefly explain what your company or institution produces, provides, or is responsible for within animal, nutrition, or environmental systems.
2. Use of mathematical and computational models
  How does your organization use mathematical or computational models (e.g., nutrition, production, environmental, or economic models) to support decision-making, operational planning, or performance improvement?
3. Integration of precision livestock technologies
  In what ways do you use precision livestock technologies and associated data platforms, models, applications, or external consultants to support modeling, prediction, or management decisions?
4. Challenges and opportunities
  What do you see as the major challenges and opportunities associated with the expansion of models, AI, and advanced technologies for strengthening animal production and sustainability?
5. Learning, adoption, and strategic alignment
  How do you and your organization continue to learn about emerging nutrition and livestock models, and how does this ongoing learning align with infrastructure, workforce capacity, and long-term strategy?

A total of 8.5 h of interview transcripts from 11 interviewees were transcribed in Microsoft Teams™ and analyzed using a qualitative, insight-driven approach modeled after a “Harvard Business Review–style” synthesis. The analysis of the qualitative data followed procedures (Turner et al. 2013) where text was assessed for commonalities in key words or phrases related to mathematical models within the context of the five questions by the research team who conducted the interviews. The summaries of these commonalities were then reviewed in an online group setting to determine general themes. The summaries were provided to interviewees only for correctness and clarification of their statements, but not for interpretation or synthesis. Thus, we were able to identify recurring themes in how models are deployed, scaled, and trusted in practice in production settings based on industry experience. The model experience captured by this interview and synthesis process is what we sought to duplicate following the example of the Harvard Business Review™, providing examples from industry experience so others can learn from hard won successes or failures. The examples were then intertwined into this review to bring an industry “lens,” to the readers, to question mathematical models beyond standard academic practice (eg, validated models from graduate student projects that end up on a shelf collecting dust). Further, we identified major limitations across model applications and technologies to present an objective review rather than to promote or compare specific model products, services, or associated technologies. Insights were classified into six cross-cutting themes relevant to accelerating model transfer to real-world livestock production constraints which included: 1) Model Augmentation of Systems, 2) Model Predictive Control (MPC), 3) Human Centered Adoption, 4) Technology Fit, 5) Data Bottlenecks, and 6) Data Enlightened Value Chain (Figure 1).

**Figure 1:**
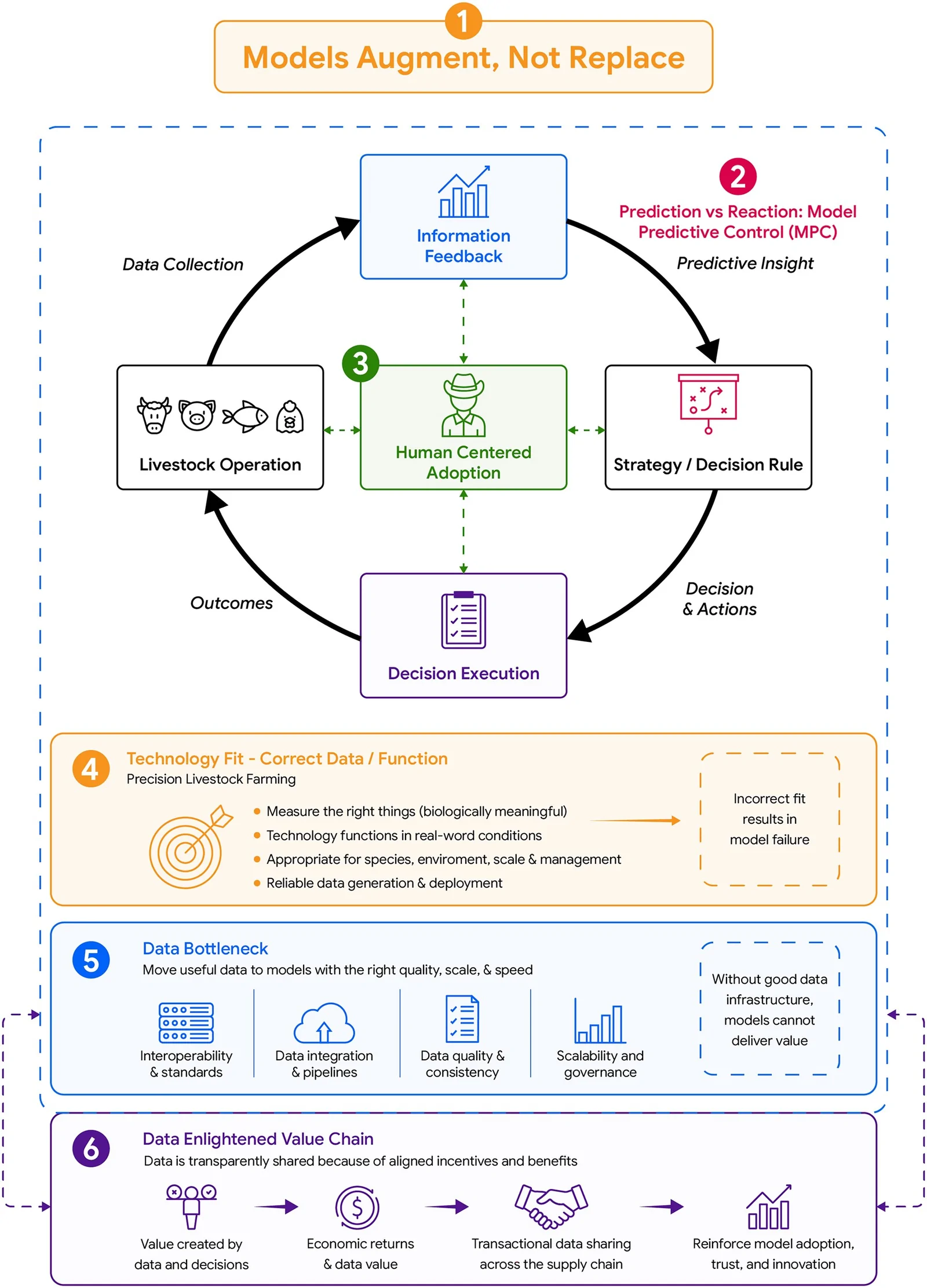
Conceptual framework describing major themes surrounding mathematical nutrition model translation into livestock feed management systems.

### Theme 1—model augmentation of systems

Across interviews, a consistent pattern emerged: the most successful livestock models were not designed to replace producers, but to augment managerial judgment under conditions of biological, operational, and economic complexity. Interviewees described models as systems that compress uncertainty, clarify trade-offs, and direct attention toward the decisions that matter most. In practice, models gained traction when they functioned less as autonomous technologies and more as operational copilots embedded within existing management workflows.

At Jorgensen Land & Cattle, this augmentation role is visible within one of the most operationally complex environments in the beef industry. Managing thousands of high-value seedstock animals requires nutritional consistency across seasons, genetics, forage systems, and production stages, creating a scale of complexity that exceeds what observation alone can reliably manage. Jorgensen described these opportunities as “rocket fuel for operations that know how to take advantage of them,” yet the operation continues to rely heavily on practical systems that support managerial interpretation rather than automate decisions outright. Models and analytics function as checkpoints that identify where attention is needed, which trade-offs matter, and when intervention is warranted, without disrupting the cadence of daily work. Crucially, modeling does not replace intuition or experience. Instead, it disciplines decision-making in systems too large to manage informally, helping experienced managers focus attention where it has the greatest leverage.

This same philosophy appeared across companies working in very different sectors of livestock production. At Halter, large-scale virtual fencing and behavioral analytics systems were intentionally designed to preserve producer agency rather than centralize authority within algorithms. Beaumont emphasized that the system exists to “support, not replace, producer decision-making,” with management judgment deliberately remaining central to the operational process. Millions of high-frequency behavioral observations are converted into grazing, rumination, and movement insights, but those insights are only valuable when they simplify decisions and translate directly into management action. Across grazing management, riparian protection, wildfire mitigation, and pasture utilization, the defining design principle was operational clarity: enabling precise decisions without overwhelming producers with unnecessary complexity.

Nofence revealed a similar dynamic from a different angle. Filbert described virtual fencing not simply as a livestock technology, but as a system that transforms fragmented observations into shared operational awareness. Decisions that once relied on delayed communication, missing animals, or isolated observations now occur collaboratively through a common view of landscape use and animal behavior. Importantly, the technology does not displace producer knowledge; it refines and stabilizes it. Heat maps and behavioral visibility challenge long-held assumptions while aligning generational knowledge and management partners around what is actually occurring on the land. As one producer described, the greatest impact was not necessarily more data, but the reallocation of time away from fence maintenance and crisis response toward higher order decisions such as grazing strategy, succession planning, and enterprise diversification.

Ontrak Nutrition demonstrated how mechanistic models become operationally valuable when tightly coupled with biological interpretation and commercial decision-making. Built on whole-animal dynamic rumen modeling originating from the University of Edinburgh, the system integrates rapid forage analysis, mechanistic simulation, and experienced nutritional consulting into a daily workflow rather than a periodic planning exercise. Holland and Ambriz-Vilchis repeatedly emphasized that the model is not self-operating; its value depends on experienced interpretation capable of translating biological outputs into practical management action. In many cases, these systems are deployed not on showcase farms, but on financially stressed operations requiring defensible explanations for why production systems are succeeding or failing. The resulting insight was clear: models gain adoption not because they are technologically sophisticated, but because they consistently explain biological outcomes and support better decisions under real constraints.

Across interviews, stakeholders consistently emphasized that operational simplicity matters more than algorithmic complexity. Producers repeatedly stated that they “want to ranch, not manage spreadsheets,” reinforcing that tools succeed when they reduce cognitive load rather than expand it. Sweeting argued that relative rankings and actionable thresholds frequently outperform absolute precision, particularly in areas such as methane selection and disease triage. Similarly, Lehr emphasized the importance of actionable outputs in commercial barns, “not big graphs,” but signals that survive real-world variability and support timely intervention. Across settings, successful systems framed outputs in operationally intuitive formats such as rankings, binary classifications, percent changes, and exception-based alerts (obvious deviations from normal vs. indicating that things are normal).

Thus, a common principle underlying successful model transfer in livestock systems emerged: models create value when they augment human systems rather than compete with them. Systems failed when they expanded the number of choices, demanded additional interpretation, or disrupted established workflows. In contrast, systems persisted when they constrained complexity, clarified action, preserved producer control, and integrated naturally into the operational rhythm of livestock production. The dominant message was not that AI or automation would replace livestock managers, but that the future of livestock systems depends on models that help experienced people make better decisions under increasingly complex conditions.

### Theme 2—model predictive control

If Theme 1 demonstrated that successful models augment human decision-making, Theme 2 revealed a deeper challenge: many livestock systems still rely on information that arrives too late to meaningfully alter biological outcomes. Across interviews, stakeholders distinguished retrospective monitoring from predictive control, arguing that future livestock systems must move beyond dashboards that describe what already happened toward systems capable of anticipating biological responses before productivity, welfare, or health decline occurs. Berckmans framed this transition most directly through the statement: “Real time is already too late—prediction grounded in the nutritional modeling-based animal energy equation is the only way to turn welfare and efficiency into the same decision.”

At the center of Berckmans’ framework is the animal energy equation, where feed energy is continuously partitioned among basal metabolism, immune activation, thermoregulation, activity, mental stress, and production. In this framing, welfare is not separate from productivity; it is fundamentally an energetic allocation problem. Heat stress, poor environments, disease, or behavioral disruption divert energy away from milk production, growth, and reproduction before visible symptoms emerge. By the time real-time alerts identify depressed intake, elevated temperature, or reduced production, substantial energetic losses have often already occurred. Berckmans therefore emphasized that livestock systems should increasingly operate through MPC, where feeding, ventilation, environmental management, and welfare interventions are adjusted proactively based on predicted responses rather than retrospective measurements.

This perspective reframes precision livestock farming from a monitoring problem into a control problem. The objective is no longer simply collecting more data, but preserving energy within productive biological pathways by anticipating disturbances before they escalate into losses. For producers and nutrition advisors, this means treating feeding and environmental management decisions as dynamic energy allocation problems where the goal is to minimize energetic “spillage” into non-productive sinks such as thermal stress, immune activation, excessive activity, or behavioral disruption. Welfare and efficiency therefore become mutually reinforcing outcomes rather than competing priorities.

Across interviews, this predictive logic repeatedly appeared in operationally pragmatic forms. Stakeholders emphasized that early-moving signals often provide more value than exhaustive retrospective analytics. Abrupt changes in water intake, feeding behavior, movement patterns, or barn-level anomalies were described as actionable triage indicators capable of surfacing biological disturbances hours before visible symptoms appear. Importantly, the usefulness of these systems depended less on generating additional dashboards and more on compressing time-to-action by creating lead time for managers to intervene. In extensive grazing systems, where animals are distributed across large landscapes and labor availability limits rapid response, interviewees emphasized that real-time alerts rarely create sufficient opportunity for intervention. Prediction is what generates operational leverage.

This requirement for anticipatory management also shaped how stakeholders discussed sensing infrastructure. Berckmans repeatedly emphasized that predictive systems only succeed when sensing technologies are designed for commercial environments rather than controlled research facilities. Sensors must survive barn conditions, operate continuously under operational variability, and generate biologically interpretable signals that can feed predictive frameworks rather than simply produce large quantities of disconnected data. In this context, sensing technologies are valuable only when tightly linked to biologically grounded predictive logic.

Smith from CERES Tag illustrated how continuous behavioral monitoring systems may support predictive livestock management by generating persistent, time-stamped datasets across commercial production environments. Rather than functioning as a decision engine itself, the system focused on maintaining continuous behavioral data streams that could support development and evaluation of predictive models. They suggest that instead of embedding proprietary control algorithms within the hardware itself, the objective is to maintain persistent behavioral datasets capable of supporting validated predictive models across operations, species, and environments. This infrastructure-first approach reflects a broader theme that emerged across interviews: predictive systems fail not because biological models are absent, but because commercial livestock systems rarely generate sufficiently continuous, interoperable, and reusable data streams to sustain anticipatory management at scale.

Collectively, interviews suggested that the next generation of livestock systems will increasingly depend on predictive frameworks capable of anticipating biological responses before they become visible production losses. Across stakeholders, the central message remained consistent: the future of precision livestock farming is not retrospective observation, but proactive biological control systems that transform sensing, nutrition, welfare, and environmental management into anticipatory decision structures grounded in animal energetics and operational reality.

### Theme 3—human-centered adoption

Predictive systems only create value when they fit the human systems responsible for acting on them. Across interviews, stakeholders repeatedly emphasized that adoption barriers are rarely driven by skepticism toward technology itself, but by labor constraints, workflow disruption, trust, time, and the realities of managing complex biological systems under operational pressure. Technologies succeed not when they maximize automation or analytical sophistication, but when they fit naturally within existing management systems and reduce the cognitive and logistical burden placed on producers. As Clark summarized directly, “The hardest constraint on model transfer is not producer skepticism—it is time, labor, and the reality of daily operations.”

This human-centered perspective fundamentally reshaped how interviewees described innovation. Filbert suggested that the most important impact of virtual fencing is not containment itself, but the transformation of management culture through shared visibility. “Our technology is not only enhancing grazing management—it is redefining grazing communities.” Virtual fencing converts uncertainty into shared operational awareness by allowing family members, neighbors, and management partners to simultaneously access real-time information on animal location and behavior. Problems that previously depended on delayed communication, missing livestock, or neighbor phone calls instead become coordinated management decisions grounded in a common operational picture. In this framing, precision livestock systems do not replace relationships between producers and landscapes; they strengthen coordination and reduce friction within human management networks.

Throughout interviews, this emphasis on operational fit repeatedly outweighed technological novelty. Beaumont described how continuous feedback loops with producers, veterinarians, integrators, and academic collaborators shape technology development far more than isolated engineering goals. There was broad enthusiasm for AI, predictive systems, and advanced analytics, but stakeholders consistently stressed that technologies requiring additional labor, duplicate workflows, or major behavioral changes rarely persist commercially. Adoption depended on a much simpler principle: tools must work for producers rather than require producers to reorganize themselves around the technology.

Interviewees also highlighted that trust emerges operationally rather than statistically. Holland described how confidence in mechanistic nutritional models was not established through validation metrics alone, but through repeated moments where the system revealed biological responses that conventional tools failed to explain. For Holland, a “lightbulb moment” occurred when a model predicted a milk-production response that was observed on farm but previously unexplained (ie, the cause of increased production was unknown). Thus, credibility emerged because the model successfully explained biological mechanisms under real-world conditions. Trust consistently developed when systems demonstrated practical usefulness within existing workflows rather than when they advertised technical sophistication.

This perspective also reshaped how stakeholders viewed the role of models and AI. Sweeting reasoned that precision diagnostics scale when they fit the way producers already work and when the value generated is obvious, shareable, and operationally meaningful. Eckberg similarly described models not as isolated analytical products, but as durable infrastructure that gradually becomes embedded within everyday management systems. In this framing, models resemble operational tools rather than temporary research outputs. Their value comes from repeated and reliable use across years, operations, and changing production environments. AI was viewed cautiously when detached from real decisions, but positively when it enhanced pattern recognition, reduced uncertainty, and strengthened confidence in producer-led decisions without eroding human agency.

Several interviewees further discussed that many agricultural technologies fail because they are designed around enterprise software logic rather than around the realities of livestock management. Producers often operate under severe time and labor constraints while simultaneously managing highly variable biological systems. As a result, expecting producers to directly manage complex interfaces, dashboards, and workflows frequently creates additional burden rather than operational value. Greater impact emerged when complexity remained behind the scenes and insights were delivered through trusted intermediaries such as Extension personnel, consultants, veterinarians, nutritionists, and conservation staff who already operate within producer decision networks.

The interviews demonstrated that adoption of models enabled by precision livestock systems is fundamentally a human-systems problem rather than a purely technological one. Technologies persist when they align with operational reality, reinforce producer agency, simplify decisions, and integrate naturally into the rhythms of daily management. The future of precision livestock farming depends less on replacing people with automation and more on designing systems that strengthen trust, coordination, and human decision-making for livestock nutrition management.

### Theme 4—technology fit

While Theme 3 emphasized that adoption depends on alignment with human workflows and management realities, interviews further demonstrated that precision livestock technologies (models or sensors) fail just as often because the technologies themselves are poorly matched to biological, environmental, and operational conditions. Across stakeholders, successful systems were consistently described not as the most technologically advanced, but as the ones capable of surviving the complexity, variability, and constraints of commercial livestock production. In practice, technology fit mattered more than technological novelty.

This philosophy was summarized directly by Beaumont: “We don’t believe in AI for the sake of AI. It has to enable a clear management action.” Technology functions within production environments where reliability, connectivity, battery life, labor constraints, and operational consistency matter as much as algorithmic sophistication. Rather than emphasizing specific machine-learning architectures or analytical frameworks, the company described a fit-for-purpose approach where technologies are evaluated primarily through their ability to support actionable grazing and livestock management decisions under commercial conditions.

Beak Analytics reinforced this same principle from the perspective of early-warning health systems. Lehr emphasized that “models only matter if they survive contact with real farms—where data are messy, biology is variable, and decisions must be immediately actionable.” Rather than focusing on highly controlled datasets or ideal experimental conditions, pipelines need to be capable of functioning within the “commercial noise” of livestock systems. Heterogeneous sensor streams, including water intake, activity patterns, and vision-based cues, are translated into leading indicators of health and production challenges that can support timely intervention before visible decline occurs. Importantly, the value of these systems was tied less to model complexity than to whether the resulting insights remained actionable within fast-moving management environments.

Berckmans extended this argument further by reframing the order of technological development itself: “biology first, then sensors, then software.” Thus, most interviewees agreed that precision livestock systems fail when sensing technologies are detached from biologically meaningful questions or when sensors are designed for laboratories rather than commercial barns and pastures. Mechanistic models and predictive systems only remain useful when they rely on interpretable biological signals such as intake dynamics, thermal load, stress proxies, and behavioral disruption that can persist under real environmental variability.

Ontrak Nutrition illustrated this reality through mechanistic nutritional models deployed within financially stressed commercial operations rather than highly controlled research systems. Holland summarized the challenge succinctly: “You can have the best model in the world, but put the wrong driver behind the wheel of the Ferrari and it’s going to crash.” In these environments, technology fit depended not on showcasing sophisticated analytics, but on whether biologically grounded models consistently explained animal responses and supported defensible interventions under operational pressure. The interviews repeatedly suggested that technologies become valuable when they function reliably within imperfect systems rather than when they perform exceptionally under idealized conditions.

Sweeting highlighted the growing emphasis on developing sensing systems that prioritize biologically interpretable and decision-relevant outputs rather than sensor precision alone, particularly for applications involving reproduction, health, and methane-related traits. Importantly, these systems were intentionally designed for harsh ranch environments characterized by dust, weather variability, cold temperatures, and intermittent connectivity. Even highly accurate technologies fail when deployment assumptions ignore environmental realities, labor availability, connectivity limitations, or timing constraints associated with livestock management.

The interviews also highlighted that technology fit extends beyond hardware into how outputs are framed for operational decisions. Several stakeholders emphasized that producers rarely need exhaustive biological detail; instead, they require signals that support immediate action. Lehr and Sweeting both described how relative rankings, binary classifications, percent changes, and exception-based alerts frequently outperform highly granular outputs in commercial settings. Similarly, Jorgensen demonstrated that spreadsheet-based tools and lightweight analytical systems can remain highly effective for operational triage and enterprise-level decision support, even when more sophisticated infrastructures exist elsewhere.

Finally, interviews consistently identified multidisciplinary integration as a major but underappreciated determinant of technology fit. Successful systems required coordination among animal scientists, engineers, behavioral specialists, economists, software developers, and Extension-oriented communicators. Yet many precision livestock companies remain heavily weighted toward engineering or software development alone. As a result, technically functional prototypes often fail to transition into trusted operational systems because they lack biological grounding, economic framing, or integration into real management environments.

### Theme 5—data bottlenecks

While Theme 4 emphasized the importance of fit-for-purpose technologies capable of functioning under commercial conditions, interviews highlighted that even effective sensing systems and validated models struggle to scale when data infrastructure remains fragmented, inconsistent, and difficult to reuse. The central limitation was rarely the absence of data itself, but the inability to transform large, heterogeneous information streams into coordinated systems that persist across projects, technologies, and production environments.

This challenge was visible even within large livestock enterprises. At Jorgensen Land & Cattle, feeding and management decisions occur at a scale where small inefficiencies compound rapidly across thousands of animals, yet the operation still relies heavily on familiar tools such as Excel and Power BI to organize rations, performance metrics, and production outcomes. Large volumes of data move continuously through the system, but integrating those data into fully closed-loop decision frameworks remains operationally difficult. Rather than automating management decisions outright, the operation uses models and analytics to pressure-test assumptions before resources are committed.

Several interviewees emphasized that this challenge extends beyond individual operations into the broader architecture of precision livestock systems. Standardized platforms such as AgriWebb™ were viewed favorably not because they maximize analytical sophistication, but because they create consistent operational foundations capable of supporting scalable analytics across multiple enterprises. This consistency reduces reinvention and allows advisory services, consultants, and producers to operate from shared data structures rather than disconnected workflows. The ability to scale depends as much on “data plumbing” as on model performance itself.

Beak Analytics described this problem through the realities of commercial livestock environments where heterogeneous sensors, missing data, inconsistent workflows, and noisy biological systems are unavoidable. High-frequency collar systems can generate millions of observations per animal per day, yet the value of these data depends entirely on whether they can be translated into operationally actionable information. Importantly, interviewees repeatedly cautioned that tool performance in pilot studies seldom translates linearly into commercial environments where biology is variable, gold standards are often absent, and management decisions occur at the group rather than individual level. Lehr summarized this challenge directly through the question: “How many animals define a sick group?” Rather than relying on clean experimental endpoints, commercial validation frequently depended on producer-defined usefulness, anomaly-to-event timelines, and the ability of systems to support timely intervention under operational tension. As a result, many consulting and deployment efforts required highly customized data pipelines built around each client’s data structures, workflows, and management constraints rather than standardized research architectures.

A persistent challenge is the repeated redevelopment of models for every new sensing platform, device, or software system. This fragmentation slows adoption, prevents accumulation of validated science, and forces researchers and producers to repeatedly rebuild analytical pipelines as technologies evolve. Interviewees pointed to examples from Australia, particularly within CSIRO-linked efforts, where independently developed behavioral and intake models were embedded within stable infrastructure layers capable of persisting across changing sensing technologies. In these systems, models became transferable operational tools rather than isolated outputs tied to individual devices or projects.

Celium Group framed this issue most directly through the statement: “The bottleneck isn’t modeling—it’s coordinated, structured data and products designed around humans, not dashboards.” Hensen described how much of the actual innovation effort in livestock and conservation systems occurs upstream, where organizations spend substantial time assembling usable datasets from fragmented spreadsheets, handwritten notes, disconnected pilot projects, and scattered cloud-storage systems. What is commonly described as “having data” often fails to produce datasets that are searchable, interoperable, or reusable across projects.

This fragmentation was also linked to broader structural problems within agricultural research systems. Across interviews, stakeholders repeatedly described a cycle where pilot studies successfully demonstrate feasibility, generate publications, or produce prototype models, yet fail to compound into durable infrastructure capable of supporting long-term learning. Data generated through a particular project is frequently difficult to discover, integrate, or reuse within subsequent efforts, leading to repeated reinvention rather than cumulative progress. Pilot success alone was therefore not viewed as evidence of commercial viability; instead, persistence depended on data continuity, interoperability, governance structures, and the ability to maintain usable datasets beyond individual grant cycles or technology generations. Interviewees noted that this challenge mirrors long-standing issues addressed by the NANP, where valuable datasets and modeling advances have historically been difficult to preserve as connected institutional knowledge over time.

Several interviewees suggested that emerging AI systems may help alleviate portions of this bottleneck, not by replacing biological models, but by transforming semi-structured information into reusable datasets. Large language models and AI-assisted pipelines were viewed as potentially valuable for organizing spreadsheets, handwritten notes, scanned records, and fragmented project documentation into searchable and quarriable information systems without requiring rigid workflows or uniform data entry structures.

Together, the interviews demonstrated that the future scalability of precision livestock systems depends not only on advances in sensing or predictive modeling, but on the development of coordinated, interoperable, and persistent data infrastructures capable of allowing validated science to accumulate across projects, technologies, and generations of livestock systems. The main message remained consistent: the primary bottleneck is increasingly not the absence of models, but the absence of durable data systems capable of carrying those models forward over time.

### Theme 6—data enlightened value chains

Interviews consistently suggested that solving interoperability and infrastructure challenges alone is insufficient for long-term adoption. Persistent data systems require not only technical continuity, but also economic. The ability of precision livestock technologies to scale depended on whether data-driven insights generated recognizable value for producers, supply-chain partners, and downstream markets simultaneously. As data systems become increasingly connected to sustainability verification, procurement programs, genetic selection, animal health, and environmental accounting, the challenge shifts from simply moving data across platforms to ensuring that value generated from those data flows transparently back through the livestock production system. In this framing, durable adoption depends not only on interoperable infrastructure, but on the development of a “data enlightened value chain” where predictive models, management decisions, and economic incentives remain aligned across producers, processors, retailers, and consumers.

Stakeholders repeatedly emphasized that producers often generate the biological and operational data underlying precision livestock systems while downstream actors capture much of the resulting value. Without mechanisms that return measurable benefit back to farms, whether through procurement premiums, sustainability incentives, reproductive gains, avoided losses, or improved operational efficiency, data collection systems to inform nutrition models are unlikely to persist. Sweeting emphasized that adoption depends on aligning economic, environmental, and social value simultaneously, particularly as sustainability programs increasingly rely on traceable and interoperable datasets linked to Scope 3 accounting, environmental verification, and supply-chain reporting.

At Edge Dairy Farmer Cooperative, Eckberg described sustainability not as a side initiative, but as an operational business system that must simultaneously function for producers, processors, and consumers. Representing the Voice of Milk Consortium, Eckberg operates at the interface of sustainability verification, policy engagement, and producer implementation across hundreds of dairy farms. Rather than treating models as isolated optimization tools, the cooperative uses whole-farm accounting systems and mechanistic models to translate producer decisions into measurable outcomes recognized across the broader value chain. Greenhouse gas mitigation, water quality, and watershed outcomes are framed not as abstract metrics, but as verifiable and economically relevant indicators linked directly to management decisions occurring at the farm level. As Eckberg summarized, “We need to better align computational models with farmer-led decision-making—so sustainability, profitability, and commercial relevance actually move together.”

This systems-oriented framing also appeared in discussions surrounding infrastructure and model deployment. Smith described a recurring challenge in livestock systems where validated models often fail to reach animals operating under real commercial conditions because sensing technologies, software systems, and deployment pathways remain fragmented. Rather than tightly coupling models to individual hardware systems, he described an infrastructure-first approach where continuous behavioral data streams could support multiple applications simultaneously, including nutrition, reproduction, welfare, and sustainability analytics. By separating validated models from individual sensing devices, stakeholders argued that the same infrastructure could support multiple layers of value creation across the supply chain without forcing producers to adopt separate technologies for each use case.

Importantly, interviewees repeatedly emphasized that measurability alone does not guarantee adoption. Precision livestock systems scale only when outputs fit seamlessly within producer workflows and when the resulting value becomes visible enough to justify participation. Several stakeholders noted that only a fraction of producers function as true early adopters actively seeking new technologies, while many others operate under retirement pressures, succession uncertainty, labor constraints, or limited incentive to alter existing systems. As a result, successful scaling depends on engaging early adopters first, demonstrating durable value under commercial conditions, and gradually allowing benefits to propagate across supply-chain relationships through trusted networks and observed outcomes rather than through top-down mandates or technological enthusiasm alone.

Others illustrated how this process unfolds operationally when growth is guided by producer trust, management fit, and clearly defined outcomes rather than by technological expansion. Technological maturity is not defined by the quantity of sensors or algorithms deployed, but by how effectively systems integrate into existing management structures while strengthening confidence across producers, advisors, and supply-chain partners. Precision livestock technology systems, predictive analytics, sustainability verification, and feed management increasingly function as connective infrastructure linking previously disconnected actors through shared operational visibility and measurable outcomes.

Interviewees further claimed that the future of predictive livestock systems depends on building coordinated ecosystems rather than isolated technologies. Researchers, funders, processors, policymakers, and technology developers all play roles in determining whether predictive feed management systems persist beyond pilot studies. Especially when incentive structures linked to procurement, sustainability verification, and environmental accounting were viewed as increasingly important mechanisms for rewarding producers for maintaining animals within biologically favorable states that improve both welfare and productivity.

Collectively, the interviews demonstrated that the long-term success of precision livestock systems depends not only on predictive accuracy or sensing capability, but on whether value generated from biological data can circulate transparently and persistently throughout the livestock production system. The message remained consistent: models become durable when they function not as isolated analytical outputs, but as shared infrastructure supporting trust, coordination, sustainability, and economically meaningful decision-making across the entire value chain.

### Limitations of models and technology for nutrition systems

The rapid advancement of nutrition models warrants careful consideration to avoid unforeseen or unintended consequences such as model failure, cybersecurity, data privacy, and environmental cost of technology such as hardware waste (batteries) and plastics entering food production systems (Kaniyamattam et al. 2026). Further, despite decades of investment risks incurred by the Agri tech industry and excitement surrounding models enabled by precision livestock technologies, relatively few technologies achieve widespread long-term adoption in commercial feeding systems. Many systems fail operationally due to labor demands, maintenance challenges, data limitations, infrastructure constraints, or lack of integration into real-world decision-making (Menendez et al. 2022, 2026). Even fewer technologies demonstrate a consistent and measurable return on investment for producers once subscription costs, replacement cycles, labor, and management complexity are considered (Hoag et al. 2025; Wongpiyabovorn et al. 2025). As technologies enable precision-informed nutrition models to become more common, producers and operations are increasingly expected to become data managers and technology integrators in addition to livestock managers. Further, if predictive models and AI systems become universally available, competitive advantages derived solely from access to technology may diminish over time (Barney and Reeves 2024). In that future, differentiation will depend less on possessing technology and more on the ability to interpret complex systems, anticipate unintended consequences, and adapt faster than competitors within rapidly changing agricultural environments.

Systems thinkers are uniquely positioned to maintain a competitive advantage in this emerging landscape because they understand interactions, feedback loops, delays, adaptation, and system-level tradeoffs rather than isolated variables (Turner et al. 2016). Future agricultural systems will likely integrate AI, autonomous sensing, large-scale optimization, and eventually quantum computing capabilities that can rapidly process highly interconnected biological and environmental datasets (Tedeschi 2026). However, these tools still require individuals capable of framing the right questions, identifying leverage points, and understanding how changes propagate through complex agricultural systems. For example, systems thinkers can integrate technology, AI, economics, ecology, and producer behavior into unified decision frameworks rather than treating technologies as isolated products. This creates a sustained advantage because the ability to anticipate change and model system behavior becomes more valuable than technology or model innovation itself.

## Conclusions

Mathematical modeling in animal nutrition will have its greatest impact when biology, data, decision models, and human systems operate in concert. Our review and interviews show that progress hinges less on inventing new algorithms and more on operationalizing prediction, grounding decisions in the animal energy equation, feeding models with trustworthy and interoperable data, and delivering outputs that match the tempo and constraints of commercial livestock systems. Precision technologies are most effective as enablers when they are fit for purpose, modular, and deployed through open data pipelines that return value to producers and align incentives across the value chain. In practice, this requires moving beyond real-time dashboards toward MPC, designing decision support around labor and workflow constraints, and evaluating success under farm-level variability rather than laboratory idealism. It also requires investment in multidisciplinary, Extension-style teams that translate validated science into repeatable action on animals and landscapes. The interviews suggest that this translation function is increasingly carried out by small, flexible expert teams operating across organizations, rather than by standalone technologies or single institutions.

For the NANP, these findings indicate that the next phase of leadership is not only centered on developing additional models, but on enabling their coordinated use, reuse, and integration across projects, regions, and decision contexts. The NANP Modeling Committee and Feed Management Committee are uniquely positioned to advance this transition by aligning biological realism, data standards, and incentive structures with the USDA’s strategic directives emphasizing productivity, resilience, stewardship, and food security. This role extends beyond scientific innovation to stewardship of data continuity, institutional memory, and trust in model-supported decisions. Taken together, the central judgment of this work is that the limiting factor for impact in animal nutrition modeling is no longer solely scientific capability, but integration. Models that are predictive, interoperable, and embedded in human decision systems persist and scale, while those that remain isolated, opaque, or misaligned with operational realities do not. The future influence of modeling within livestock systems will therefore be determined not by the sophistication of individual algorithms, but by the collective ability to coordinate their use in service of producers making decisions under real-world constraints.

## Abbreviations

AI: artificial intelligence
MPC: model predictive control
NANP: National Animal Nutrition Program
NRC: National Research Council
